# Effects of Reactive Oxygen Species on Tubular Transport along the Nephron

**DOI:** 10.3390/antiox6020023

**Published:** 2017-03-23

**Authors:** Agustin Gonzalez-Vicente, Jeffrey L. Garvin

**Affiliations:** 1 Department of Physiology and Biophysics, School of Medicine, Case Western Reserve University, Cleveland, OH 44106, USA; jlg5@case.edu; 2 Facultad de Farmacia y Bioquímica, Universidad de Buenos Aires, Ciudad Autónoma de Buenos Aires C1113AAD, Argentina

**Keywords:** renal physiology, water homeostasis, epithelial transport, salt-sensitive hypertension

## Abstract

Reactive oxygen species (ROS) are oxygen-containing molecules naturally occurring in both inorganic and biological chemical systems. Due to their high reactivity and potentially damaging effects to biomolecules, cells express a battery of enzymes to rapidly metabolize them to innocuous intermediaries. Initially, ROS were considered by biologists as dangerous byproducts of respiration capable of causing oxidative stress, a condition in which overproduction of ROS leads to a reduction in protective molecules and enzymes and consequent damage to lipids, proteins, and DNA. In fact, ROS are used by immune systems to kill virus and bacteria, causing inflammation and local tissue damage. Today, we know that the functions of ROS are not so limited, and that they also act as signaling molecules mediating processes as diverse as gene expression, mechanosensation, and epithelial transport. In the kidney, ROS such as nitric oxide (NO), superoxide (O_2_^−^), and their derivative molecules hydrogen peroxide (H_2_O_2_) and peroxynitrite (ONO_2_^−^) regulate solute and water reabsorption, which is vital to maintain electrolyte homeostasis and extracellular fluid volume. This article reviews the effects of NO, O_2_^−^, ONO_2_^−^, and H_2_O_2_ on water and electrolyte reabsorption in proximal tubules, thick ascending limbs, and collecting ducts, and the effects of NO and O_2_^−^ in the macula densa on tubuloglomerular feedback.

## 1. Introduction

Reactive oxygen species (ROS) are by definition oxygen containing molecules whose interactions with other compounds are energetically favorable. ROS include oxygen molecular allotropes, such as singlet or triplet forms, as well as oxygen in combination with other atoms; however, this article focuses on the effects of NO, O_2_^−^, and their derivative molecules H_2_O_2_ and ONO_2_^−^ on water and electrolyte reabsorption along the renal nephron, as well as on their ability to regulate tubuloglomerular feedback. The effects of ROS on other aspects of renal physiology and pathology have been reviewed elsewhere [1,2]. 

## 2. Proximal Tubule

The proximal tubule reabsorbs 50–60% of the filtered inorganic solutes, and nearly all of the sugars and amino acids. Na extrusion from the cell by the basolateral Na/K-ATPase provides the driving force for passive Na entry across the apical membrane as well as for all other transport processes. Na enters the cell mainly via the electroneutral Na/H exchanger (NHE). Na/H exchange by NHE3 facilitates bicarbonate reabsorption due to the presence of carbonic anhydrase in the luminal membrane. As the luminal bicarbonate concentration decreases, NaCl is reabsorbed due to NHE3 and Cl/bicarbonate exchange. The Na gradient is also used by several cotransporters to drive glucose, phosphate, amino acids, and other components of the ultrafiltrate into the cell. The proximal tubule reabsorbs water isoosmotically so that Na and fluid reabsorption can be equated. Most water traverses the cells via aquaporin-1 (AQP-1) channels constitutively expressed in both the apical and the basolateral membranes. The remaining water is reabsorbed via the paracellular pathway. Several ions move with this water due to solvent drag. 

### 2.1. NO and ONO_2_^−^

**Figure 1:**
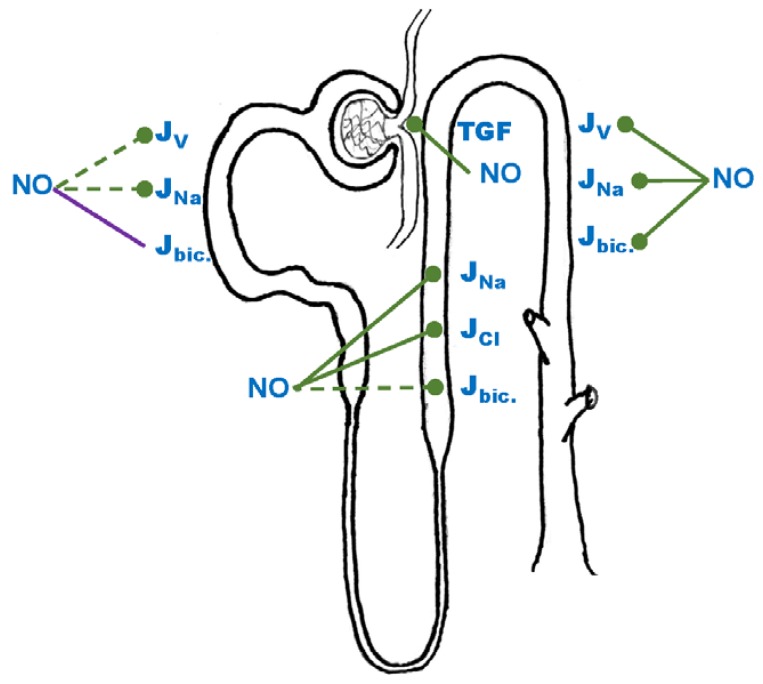
Effects of nitric oxide (NO) on net fluid reabsorption (J_v_), sodium reabsorption (J_Na_), bicarbonate reabsorption (J_bic._) along the nephron and on tubuloglomerular feedback (TGF). Blunted green line: inhibition, purple line: unknown effect, dashed line: some conflicting or missing data.

Most studies indicate that NO reduces fluid and Na reabsorption in this segment (Figure 1). Lithium clearance, a measure of proximal tubule Na reabsorption, is blunted by NOS inhibition [3]. These data indicate that NO inhibits Na transport in this segment in vivo. Micropuncture studies in rats indicate that the NO donor nitroprusside reduces proximal nephron net fluid reabsorption (J_v_) by 23% or 35%, when added to the luminal or peritubular perfusate respectively [4]. Consistent with an inhibitory effect of NO in proximal tubule transport, intratubular addition of L-nitroarginine methylester (L-NAME) to rat micropunctured proximal tubules increased J_v_ by 17% [5]. The stimulatory effect of L-NAME and low angiotensin II (Ang II) concentrations are blunted by NO donors [4,5]. Similarly, L-NAME blunts the larger response in Na/K-ATPase activity to low Ang II concentrations in proximal tubules from spontaneously hypertensive rats (SHR) as compared to Wistar-Kyoto (WKY) [6]. On the other end, the inhibitory effect of high Ang II concentrations on Na/K-ATPase and NHE are mediated by NO [7]. The inhibitory effect of NO on Na reabsorption by proximal tubules is likely due to a reduction in NHE3 activity. NO donors reduce NHE activity in primary culture of proximal tubule cells [3] and cGMP, the second messenger of NO, appeared to mediate these effects in both freshly isolated proximal tubules and in cultured cells [3].

The effects of NO on proximal tubule transport could be caused by diffusion of NO from surrounding vessels or from NO produced in the proximal tubules themselves. NO synthase (NOS) 1 (-/-) mice have higher fluid and chlorine reabsorption rates than those isolated from their wild type mates, indicating that NO acts as an autacoid [8]. Similarly, treatment of proximal tubule cells with cytokines to stimulate NO production by NOS2 has been reported to inhibit Na/K-ATPase activity in an NO-dependent manner [9]. However, it is likely that this effect was due to ONO_2_^−^ rather than NO itself as it was blunted by O_2_^−^ scavengers and it has been shown that high concentrations angiotensin II inhibit the pump via ONO_2_^−^ [10]. Furthermore, ONO_2_^−^ rather than NO inhibits Na/K-ATPase in other nephron segments [11].

Although the bulk of evidence indicate that NO exerts inhibitory effects on transport in proximal tubules, there are reports to the contrary (Figure 1). High concentrations of NO stimulate transport [12]. Fluid reabsorption as measured by micropuncture was reduced by intratubular infusion of L-NAME, as well as by specific inhibitors of NOS1 and NOS2 in one report [13]. In addition NOS1 (-/-) and NOS2 (-/-) mice have been reported to have lower PT reabsorption rates in at least two studies [14,15] than their wildtype mates. The explanations for the disparate data are unclear. However, in micropuncture and in-vivo studies, factors that alter GFR can affect proximal tubule Na reabsorption through glomerular-tubular balance [16,17]. Thus, the effects of inhibitors on either the renal vasculature or downstream structures may indirectly alter proximal tubule function. Potential explanations for the disparate data in (-/-) mice are different. For data obtained from NOS1 (-/-) mice, it must be recognized that the early generations of NOS1 mice had a deletion in the gene that only disrupted expression of NOS1α but other splice variants did not. Thus the opposing results may be due to different NOS1 splice variants being knocked out in different tissues. Alternatively, NO may have biphasic actions on transport by this segment, with low concentrations having an inhibitory effect and high concentrations a stimulatory one. Finally, the actions of NO may not be all due to NO itself but to its adducts. The best known of these is ONO_2_^−^, but NO can also react with thiol and other groups producing many distinct end products that may alter proximal nephron function differentially. Further studies should investigate the sources and concentrations of NO as an important determinant of its actions.

### 2.2. O_2_^−^ and H_2_O_2_

**Figure 2:**
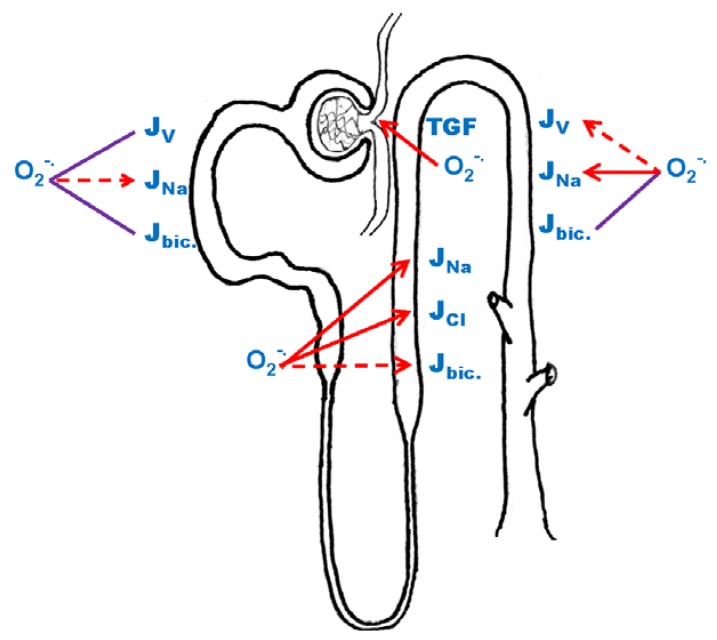
Effects of superoxide anion (O_2_^-•^) on net fluid reabsorption (J_v_), sodium reabsorption (J_Na_), bicarbonate reabsorption (J_bic._) along the nephron and on tubuloglomerular feedback (TGF). Red arrow: stimulation, purple line: unknown effect, dashed line: some conflicting or missing data.

The effects of O_2_^−^ and H_2_O_2_ on proximal tubule function are poorly understood (Figure 2). Studies conducted in spontaneously hypertensive rats (SHR), a model characterized by high oxidative stress levels in the kidney suggest that O_2_^−^ reduces Na reabsorption. Measurement of J_v_ by micropuncture showed that adult hypertensive SHR have lower rates than the normotensive control Wistar-Kyoto (WKY) rats [18]. By using siRNA and inhibitors of NADPH oxidase (NOX) and O_2_^−^ scavenger TEMPOL, it was demonstrated that NOX-derived O_2_^−^ was responsible for the blunted transport activity in SHR, while they had no effect on J_v_ in WKY rats [18]. 

The results reported above contrast with data from diabetic animals (a pathological model) also characterized by high oxidative stress levels. In the early stages of diabetes, proximal tubule fluid reabsorption is elevated [19]. This elevation, appears to be due to an increase in O_2_^−^ production since NADPH inhibition reduces Na transport [19]. Thus, unlike in the SHR studies, in diabetes O_2_^−^ stimulates Na reabsorption by proximal tubules. 

The explanation for the apparent differential effects of O_2_^−^ on Na reabsorption in the proximal tubule are difficult to explain. One possibility is that the enhanced O_2_^−^ in diabetes acts differently than the elevated levels of O_2_^−^ found in SHR. An alternative is that, like NO, the effects of O_2_^−^ are biphasic and different concentrations of O_2_^−^ are produced in SHR and diabetic rats. Finally the differences between results may have to do with varying levels of H_2_O_2_ rather than O_2_^−^ itself. In this regard, increased H_2_O_2_ production and advanced glycation end products in response to high glucose in cultured cells inhibits the Na/glucose cotransporter [20]. Additionally, H_2_O_2_ inhibits Na/K-ATPase in cultured mouse proximal tubule cells [9].

NOX4 is the likely primary source of O_2_^−^ in the proximal tubule [21,22]. In cultured proximal tubule cells, NOX4 is responsible the enhanced O_2_^−^ production in response to glucose [23]. NOX4, but not NOX1 or NOX2, expression is elevated in the renal cortex of diabetic mice and mouse proximal tubule cells exposed to high glucose [23]. In addition, NOX4 down regulation by targeted siRNA or inhibition by GK136901 (NOX4 and 1 specific inhibitor) in proximal tubule cells, attenuated glucose-mediated increases in ROS generation [23].

## 3. Loop of Henle

Thick ascending limbs reabsorb about 25–30% of the NaCl load filtered through the glomerulus. As in the proximal nephron, basolateral extrusion of Na by the Na/K-ATPase drives all cellular transport processes. The bulk of Na, about 70–80%, enters the cell via the apical Na/K/2Cl cotransporter (NKCC2). Cl exits via the basolateral Cl channels and K-Cl cotransport, while K exits the cell via both luminal and basolateral K channels. Thick ascending limbs also express carbonic anhydrase which allows reabsorption of most of the bicarbonate that escapes the proximal tubule. This process is associated with proton extrusion by NHE3 which accounts for ~25% of the Na reabsorbed by this segment. As opposed to the proximal tubule, the thick ascending limb is impermeant to water. Ca and Mg reabsorption occurs passively via the paracellular pathway driven by the positive luminal voltage.

### 3.1. NO and ONO_2_^−^

NO is a major factor in the regulation of transport by the thick ascending limb, and there is general agreement in the literature on its effects (Figure 1). The ability of NO to inhibit transport in this segment was first shown by a study in isolated, perfused rat thick ascending limbs where addition of the NO-donor spermine-NONOate [24] reduced net NaCl reabsorption. Subsequently, it was shown that stimulation of NO production with the NOS substrate L-arginine [25,26] decrease net Cl absorption (J_Cl_) [24,25] and bicarbonate reabsorption [26]. The effects of L-arginine on NO and transport, are blocked by the general NOS inhibitor L-NAME [26,27]. Taken together, these data indicate that endogenously produced NO inhibits transport by the thick ascending limb; however, they do not identify the source. 

The mechanism by which NO inhibits NaCl reabsorption is well-established. Experiments measuring the initial rate of intracellular Na increase in isolated-perfuse tubules, when switching from a Na-free to a Na-containing perfusion solution, showed that NO decreases apical Na entry in the rat thick limb. This effect was attributed to a decrease in NKCC2 activity, since it remains in the presence of the NHE3 blocker dimethyl amiloride. The authors also concluded that it was not due to inhibition of luminal K channels because valinomycin added to the luminal perfusate did not blunt the inhibition produced by NO [28]. The cause of the reduction in NKCC2 activity is a decrease in the number of transport proteins in the luminal membrane. NO decreases trafficking of NKCC2 to the luminal membrane and increases its retrieval [29,30,31].

NO also affects luminal K channels, but this is likely in the opposite direction, i.e., a stimulatory effect. Cell-attached patch clamp experiments indicate that NO stimulates the luminal 70-pS K channel activity in the rat thick ascending limb [32]. In contrast, measurements of membrane voltage show that NO does not alter changes in voltage induced by switching luminal K from 1 mM to 25 mM. From these data, the authors concluded that NO does not affect luminal K channels [28]. It is likely that this conclusion was incorrect because K channels generate the only conductance in the luminal membrane. Thus, this experimental design was actually not adequate to test whether NO increases, decreases, or has no effect on luminal K channel activity. Similarly, the valinomycin experiments cited in the preceding paragraph test whether NO decreases K conductance, but do not provide evidence for a positive effect of NO. However, valinomycin experiments suggest that increases in luminal membrane K conductance as found in patch clamp experiments to not increase net NaCl reabsorption.

The effects of NO on NHE3 were studied by measuring pH recovery upon application of an acid load in perfused tubules. In this preparation, NO donors inhibit both apical and basolateral Na/H exchanger activity [33]. These results are consistent with others obtained in caco-2 cells, where NO/cGMP pathway inhibits NHE3 activity [34]. Thus there is consistent data showing that NO inhibits NHE3 in thick ascending limbs. That said, Na/H exchange is linked to HCO_3_^−^ membrane voltage reabsorption [35], and the effects of NO on bicarbonate reabsorption remain to be clarified. On the one hand (and in good agreement with the inhibitory effects of NO on Na/H exchange), direct measurement of bicarbonate reabsorption in isolated perfused tubules showed an inhibitory effect of NO [26], but it has also been reported that stimulation of endogenous NO by L-arginine or addition of NO donors increases bicarbonate transport [36]. Interestingly, similar animals, buffers, and techniques were used in both reports, and further research is required to explain this discrepancy (Figure 1).

Finally, the effects of NO on Na/K-ATPase were studied by measuring changes in transport related oxygen consumption by equalizing intracellular Na at different levels. In these experiments, NO did not affect maximal turnover or the affinity of Na/K-ATPase for Na [28]. Consistently, inhibition of cGMP-dependent protein kinases, did not affect Na/K-ATPase maximal activity or affinity for sodium in microdisected thick ascending limbs [37]. This data indicate that NO has no direct effect on Na/K-ATPase; however, in the presence of O_2_^−^, NO forms ONO_2_^−^, which inhibits the pump [9,11].

In thick ascending limbs, NO acts primarily by stimulating soluble guanylate cyclase and increasing cGMP [32,38], but after this step the signaling pathway for the inhibition of NaCl and Na bicarbonate differ. Cell permeant analogues of cGMP mimic the effects of NO on NaCl reabsorption [26,39,40,41]. Inhibitors of cGMP-stimulated phosphodiesterase, but not cGMP-dependent protein kinase, block the effects of NO on NaCl reabsorption, and low levels of an analogue of cAMP resistant to hydrolysis blocks the effects of NO. From these data, it was concluded that NO activates soluble guanylate cyclase and increases cGMP. cGMP, in turn, activates a phosphodiesterase that degrades cAMP. The reduction in cAMP reduces cAMP-dependent protein kinase activity and this reduces the number of NKCC2 transporters in the luminal membrane [29,31,42]. In contrast, bicarbonate reabsorption and presumeably NHE3 activity is inhibited by an increase in cGMP and activation of cGMP-dependent protein kinase. The steps beyond this kinase are currently unknown. 

The thick ascending limb expresses all three isoforms of NO synthases (NOS1, 2 and 3), and L-arginine decreases transport in wild type mice [43] similar to what it does in rats. This effect is preserved in NOS1 or NOS2 knockout (-/-) mice, but is blunted in NOS3 (-/-) mice [43,44]. Furthermore, the inhibitory effect of L-arginine can be restored in NOS3 (-/-) mice by transducing their thick ascending limbs with a vector containing NOS3 [44]. These data indicate that NO from NOS3 but not NOS1 or NOS2 regulates transport by this segment.

### 3.2. O_2_^−^ and H_2_O_2_

The renal medulla is the primary source of O_2_^−^ in the kidney [45]. The majority of the O_2_^−^ in the thick limbs is produced by NADPH oxidases. However, a significant amount comes from sources such as the mitochondria or uncoupled NOS [46]. The effects of O_2_^−^ on transport are two-fold. On the one hand, it stimulates NaCl transport by reducing NO bioavailability [27]; on the other, it acts by enhancing transport in an NO-independent manner [47] via PKCα activation [48].

O_2_^−^ stimulates net NaCl reabsorption [49] (Figure 2) and scavenging O_2_^−^ with TEMPOL reduces transport [49]. The increase is due to O_2_^−^ augmenting NKCC2 activity as measured by furosemide-sensitive increases in intracellular Na caused by switching luminal perfusate from a KCl-free solution to one containing KCl [47]. O_2_^−^ also stimulates luminal NHE activity while it inhibits basolateral NHE activity [50]. The explanation for this difference may be that the NHE in the apical membrane is type 3, while that on the basolateral membrane is type 1, but this remains to be clarified. In addition, although the effects of O_2_^−^ on NHE activity have been studied in some detail, the effect on bicarbonate transport has not been studied yet (Figure 2). Finally, the effects of O_2_^−^ and H_2_O_2_ on aspects of transport have been partially studied and require further exploration. Na/K ATPase activity was not acutely affected by O_2_^−^ generated using hypoxanthine/xanthine oxidase [47]. In the same report, O_2_^−^ caused a mild inhibition on apical membrane K conductance [47]. Additionally, H_2_O_2_ did not alter net NaCl reabsorption but the concentration tested may have been subphysiological [49]. 

Physiological stimuli such as luminal flow [51] and angiotensin II [52] increased transport in the thick ascending limb acting through a PKC-dependent activation of NADPH oxidase and O_2_^−^ production [53,54]. Even though there is consensus around the stimulatory effect of NADPH-derived O_2_^−^ on transport, the main NADPH oxidase isoform involved is still matter of debate. NOX2 and its subunit p47_phox_ were originally identified as the main source of O_2_^−^ in this segment [55], and disruption of the gene for p67_phox_, another NOX2 subunit, decreased medullary NADPH oxidase-dependent O_2_^−^ production and attenuated salt-sensitive hypertension in Dahl salt-sensitive rats [56]. On the hand, flow [57] and Ang II-induced [53,58] production of O_2_^−^ in the thick ascending limb was reported to depend on NOX4 but not NOX2 as studied by using siRNA and (-/-) mice. Part of the disparate data may be due to the limitations of our measuring techniques, which only measure overall O_2_^−^ availability. Adding complexity, the redundancy of the cascade O_2_^−^/PKC/NADPH/O_2_^−^ [46,53] suggest that the regulatory pathways of different NADPH oxidases are not independent of each other; thus, inhibiting one of them can affect parallel pathways, leading to an overestimation of its contribution. 

## 4. Macula Densa

The macula densa is a specialized plaque of cells located at the end of the thick ascending limb where it transitions to the distal convoluted tubule. It is usually adjacent to the afferent and efferent arterioles. The macula densa cells sense luminal NaCl concentrations, such that increases in Na delivery initiate a tubuloglomerular feedback (TGF) response. During TGF a signal is generated by the macula densa that causes constriction of the afferent arteriole, thereby reducing glomerular filtration rate. The sensing mechanism is trigger by a Na-driven increase in NKCC2 cotransport causing depolarization of the macula densa cells, entry of Ca and release of ATP.

### 4.1. NO

In perfused macula densas, increases in luminal NaCl, fluid flow or viscosity stimulate NO production [59,60]. The increase in NO caused by elevated luminal NaCl is mediated by a NHE-induced increase in intracellular pH. Treatment with dimethyl amiloride blunts both the change in pH and increase in NO [59]. Flow-induced NO generation is due to a different mechanism given that it remains present when tubules are perfused with electrolytes-free dextran solutions [60]. In both cases, the increase in NO is associated with a reduction in the TGF response (Figure 1) that was blocked by the NOS1 selective inhibitor 7-NI [59,60]. Infusion of 7-NI blunts TGF only when it is infused in the tubular lumen, not in the afferent arteriole [61], indicating that the effects of NO are within the macula densa. It is currently unknown how NO/cGMP inhibits TGF but it is thought to involve NKCC2. Experiments using a mouse macula densa cell line (MMDD1) suggest that arachidonic acid metabolites may be involved [62]. This would be a different mechanism than that activated in the thick ascending limb. Finally, the interesting question of how Na/H exchange- and flow-dependent NO production interact in-vivo remains open and deserves further attention.

### 4.2. O_2_^−^ and H_2_O_2_

O_2_^−^ enhances TGF (Figure 2) by constricting the afferent arteriole and by scavenging NO at the macula densa [63]; however, the mechanism by which O_2_^−^ causes the constriction is unknown. It could be due to O_2_^−^ enhancing NKCC2 activity, some other part of the TGF signaling cascade, or affecting the afferent arteriole itself. Experiments using MMDD1 cells or rabbit isolated-perfused macula densas showed that the bulk of O_2_^−^ induced by increasing luminal salt was blunted by the NADPH oxidase inhibitor apocynin, suggesting NADPH oxidases, instead of xanthine oxidase or cyclooxygenases, as the main source of O_2_^−^ in the macula densa [64,65,66]. In addition, RTPCR conducted on rat single macula densa cells captured by laser microdissection show that the rat macula densa expresses NOX2 and NOX4 but not NOX1 [64]. A similar expression pattern was found in MMDD1 where blocking NOX2 but not NOX4 with siRNA inhibited high salt induced O_2_^−^ production [64]. Taken together, these data suggest that NOX2 is the primary source of O_2_^−^ production in macula densa cells in response to luminal NaCl.

## 5. Collecting Ducts

The collecting duct reabsorbs up to 5% of the Na load filtered through the glomerulus. Collecting ducts present two well differentiated cell types, principal and intercalated. In principal cells reabsorption of Na is linked to K secretion. Na enters the cell via the apical epithelial Na channel (ENaC). The energy for Na reabsorption is provided by the Na/K-ATPase expressed on the basolateral membrane. Na-entry into the cell generates a lumen-negative voltage which provides the energy for K secretion via apical K channels. In these cells Cl secretion is mediated by the cystic fibrosis transmembrane regulator (CFTR). Principal cells also constitutively express AQP-3 and AQP-4 in the basolateral membrane, while the expression of AQP2 in the apical membrane regulated by antidiuretic hormone (ADH). In the absence of ADH, water permeability in isolated perfused tubules is very low and J_v_ is negligible. The addition of ADH causes the insertion of AQP2 in the luminal membrane, thereby increasing J_v_ [67]. In addition, ADH stimulates urea transport in inner medullary collecting ducts [68].

The intercalated cells are involved in regulation of acid-basic balance. Type A intercalated cells secrete protons into the urine by both H-ATPase and H/K-ATPase expressed in the luminal membrane. Excretion of protons results in the intracellular formation of bicarbonate, which is exported from the cell by basolateral by anion exchangers. Type B intercalated cells express the Cl/HCO_3_ exchanger pendrin on their apical membrane allowing them to secrete bicarbonate into the lumen. 

### 5.1. NO and ONO_2_^−^

The very first direct demonstration that NO could regulate transport in any nephron came from data showing that Na reabsorption by the M1 mouse cortical collecting duct cell line was inhibited by a NO donor and this was due to cGMP generation [69,70]. This was followed by data in isolated, perfused cortical collecting ducts showing NO donors and NO released from endothelial cells inhibited net Na reabsorption (Figure 1). Since essentially all Na reabsorption was inhibited by amiloride in these studies and NO decreased intracellular Na, the authors concluded that NO inhibits Na reabsorption via actions on ENaC [71]. Single-channel patch clamp experiments on cultured amphibian distal tubule principal cells confirmed these studies and showed that NO decreased ENaC open probability [72]. As in other segments, Na/K-ATPase activity was unaffected by acute NO treatment in perfused cortical collecting ducts [71] and cultured collecting duct cells [70].

In addition to ENaC, NO generated by NO-donors have been reported to affect basolateral K channels. These results are controversial with both inhibitory [73] and stimulatory [73,74] effects reported within a narrow range of NO-donors concentrations. Even though a clear explanation of these results remains elusive, some of the results may be due to generation of ONO_2_^−^, as they could be prevented by scavenging O_2_^−^ [73,75]. Alternatively, NO may exerts biphasic effects on K channel activity, or some molecules used as NO donors may interact with K channels independently of NO [76].

Similar to the effects of K channels, the extent to which the NO/cGMP pathway regulates water reabsorption by collecting ducts is still a matter of debate. Direct measurements of fluid reabsorption in isolated-perfused collecting ducts using NO donors indicate that NO decreases water permeability [77,78] (Figure 1). This effect did not appear to be due to a direct effect on AQP2 but rather the upstream signaling cascade. Activation of cGMP-dependent protein kinase by NO decreased ADH-stimulated cAMP [78] and the effects of NO on ADH-stimulated water permeability are blunted by inhibition of guanylate cyclase and cGMP-dependent protein kinase [77]. Finally it appears that the ability of NO to reduce ADH-stimulated water permeability was due to activation of a phosphodiesterase because the non-selective phosphodiesterase inhibitor isobutyl methyl xanthine prevented NO from reducing ADH-stimulated cAMP accumulation [79]. Taken together, these data indicate that the NO/cGMP pathway modulates ADH signaling by acting on cAMP-phosphodiesterases. This signaling cascade is reminiscent of that whereby NO reduces NKCC2 activity in thick ascending limbs.

In contrast, studies using confocal microscopy of kidney slices provided evidence that the NO donor Na nitroprusside or the NOS substrate L-arginine increase the translocation of AQP2 to the plasma membrane [80]. These data suggest that NO augments water permeability and fluid reabsorption. However, results from kidney slices have largely been discredited due to concerns about tissue hypoxia, lack of luminal flow, tissue pH problems due to slow diffusion of CO_2_, and reductions in metabolic substrate availability due to limited diffusion. 

Overall, direct measurement of transport support the theory that NO directly inhibits water permeability in the collecting duct; however, concomitant actions cannot be excluded. For instance, NO-stimulated insertion or retrieval of AQP2 might depend on other factors such as osmotic gradient or initial cAMP levels or NO may inhibit the channels themselves independently of their trafficking. Further studies are required to identify the signaling cascades that led to AQP2 regulation by NO in order to differentiate direct and secondary effects.

Cortical collecting ducts not only reabsorb water and Na from the forming urine but also are responsible for fine-tuning urine pH through either secretion of protons or bicarbonate. These processes occur in intercalated cells. In freshly isolated cortical collecting ducts, NO donors decrease H-ATPase hydrolytic activity in a dose-dependent fashion [81]. This effect was mimicked by both inductors of NOS2 and cGMP analogs [81], suggesting that inducible NOS may participate in pH regulation by this segment through cGMP. In addition, activation of AT2 in mice, reduces pendrin expression in whole kidney homogenates without affecting its subcellular localization [82]. Administration of L-NAME to the animals blunts this effect, strongly suggesting NO as a downstream mediator of AT2s [82]. Further, experiments conducted in primary cultures of mouse cortical collecting ducts exposed to NO donors showed that NO regulates pendrin expression by enhancing cAMP degradation [83]. (Figure 1)

### 5.2. O_2_^−^ and H_2_O_2_

Most of the experimental data regarding the regulation of ENaC by O_2_^−^ and H_2_O_2_ come from experiments conducted in the amphibian kidney cell line A6. This cell line resembles collecting duct principal cells as far as regulation of ENaC goes. In these cells aldosterone increased ENaC activity via generation of O_2_^−^ [84] (Figure 2). In addition, insulin stimulates ENaC activity via NADPH oxidase-dependent activation of phosphatidylinositol-3 kinase (PI3K) [85]. Upon insulin stimulation, however, O_2_^−^ does not directly mediate PI3K activation, instead H_2_O_2_ was responsible for this effect [85]. Similarly, exogenous addition of H_2_O_2_ to A6 cell incubation media, increases PI3K activity and sodium transport [86].

Experiments in isolated-perfused collecting ducts show that, AT1 receptor activation by Ang II, increased ENaC activity [87]. Further, patch clamp experiments indicate that this stimulation is mediated by activation of NOX by PKC and depends on O_2_^−^ generation [88]. The positive effects of O_2_^−^ and H_2_O_2_ on ENaC activity may be, at least in part, the result of antagonizing inhibitory factors, such as arachidonic acid [88,89].

The effects of O_2_^−^ and H_2_O_2_ on basolateral K channels have been largely studied using models of dietary K deprivation or supplementation. Low K intake suppresses, whereas high K intake increases K secretion by the kidney. Low K intake is associated with increased O_2_^−^ levels in rat [90] and mice [91] kidneys. Such a diet also decreases the activity of basolateral K channels in isolated collecting ducts [90,91] as measured by patch clamp. Reductions in O_2_^−^ levels in the kidney by intraperitoneal tempol infusions increases K channel activity and urinary K excretion in rats fed a low K diet [90,91]. These data indicate that O_2_^−^ at least partially mediates the reduction in K channel activity caused by hypokalemia [91]. 

Experiments using Knockout mice lacking gp91phox-containing NADPH oxidase (NIOX2) identified this isoform as the main source of O_2_^−^ production in collecting ducts during K deprivation [91].

The increase in renal O_2_^−^ caused by hypokalemia may be due to elevated actions of angiotensin II. Rats fed a low K diet have increased AT1 receptor expression on the apical membrane of collecting ducts [92]. Angiotensin II type 1 receptor blockers blunt the increase in O_2_^−^ in the kidney [93], and this prevents inhibition of K channel activity [93].

The effect of H_2_O_2_ on K channels is not as clear as the effects of O_2_^−^. The effects of H_2_O_2_ were first studied in M1 cells where addition of 50 µmol/L to 200 µmol/L H_2_O_2_ increased c-Src, expression and phosphorylation of c-Jun [90]. These actions lead to increases in tyrosine-phosphorylation of the ROMK-like SK channel, and inhibition [92,93,94,95]. The inhibitory effect of H_2_O_2_ in ROMK-like SK channel was further explored in rat freshly isolated collecting ducts by path-clamp. In these experiments addition of H_2_O_2_ inhibited ROMK-like SK channel by internalization depending on PKT, P38 and ERK [96]. Importantly, H_2_O_2_ has no effect in conductance, when measure in inside-out patches [96], emphasizing the roll of reactive oxygen species as signaling molecules. In summary, many of the effects of O_2_^−^ are mimicked by H_2_O_2_ suggesting that the later can be a downstream mediator of the former. This, however, needs further exploration because the signaling processes leading to channel inhibition remain unknown, and single-channel activity does not necessarily represent global transport.

H_2_O_2_ not only has effects on cation transporters but also anion and organic transporters. The addition of 100 µmol/L to 300 µmol/L H_2_O_2_ to IMCD cells increases short circuit current sensitive to cystic fibrosis transmembrane conductance regulator (CFTR) inhibitors [97], suggesting that H_2_O_2_ stimulates CFTR—mediated Cl secretion. Urea transporters in inner medullary collecting ducts are also positively regulated by O_2_^−^ [98].

Data concerning participation of O_2_^−^ and H_2_O_2_ in AQP2 regulation is very limited (Figure 2). Knocking down NOX4 with siRNA in collecting ducts cell lines blunts the increase in AQP2 mRNA in response to ADH [99]. This effect was blunted by phosphodiesterases inhibitors, suggesting that O_2_^−^ enhances cAMP degradation [99]. In addition, exposure of mice kidney slices to tert-butyl hydroperoxide increases AQP2 glutathionylation [100]. Taken together these data indicate that O_2_^−^ and H_2_O_2_ participate in the regulation of AQP2, but fundamental measurements of water permeability are missing. 

## 6. Conclusions

ROS regulate salt reabsorption along the nephron as part of normal physiology. Imbalances in this regulation can lead to either salt retention or chronic salt wasting. Although there is evidence supporting a role for defective ROS signaling along the nephron in the development of hypertension in animal models, studies in humans have largely been unsuccessful. This is likely due to the fact that human hypertension develops slowly over years and the effects of ROS are confounded by many other environmental insults. Additionally, once hypertension is discovered, patients are usually treated with drugs that may overcome the effects of ROS on transport but not their actions as initiators of inflammation. This begs the questions as to how to better design animal experiments so that they are more translational and what insight can be gained from the failure to do so thus far.
